# 1684. Clinical Comparison of Pertussis Infected with Macrolides-resistance and Macrolides-sensitive Bordetella Pertussis

**DOI:** 10.1093/ofid/ofad500.1517

**Published:** 2023-11-27

**Authors:** Fengwei Guo, Jikui Deng

**Affiliations:** Division of Infectious Diseases, Shenzhen Children′s Hospital, Shenzhen, Guangdong, China; Division of Infectious Diseases, Shenzhen Children′s Hospital, Shenzhen, Guangdong, China

## Abstract

**Background:**

To analyze the clinical characteristics and therapeutic outcome of pertussis infected with macrolides-sensitive and macrolides-resistant Bordetella pertussis.

**Methods:**

The clinical data of 154 hospitalized children with pertussis diagnosed by culture in Shenzhen Children's Hospital infectious Department from December 2015 to December 2017 were collected. All children were divided into macrolides-sensitive (MS) group and macrolides-resistant group (MR) according to the results of antimicrobial susceptibility testing. The clinical characteristics and curative effect indexes were compared between these two groups.

**Results:**

From December 2015 to December 2017, 154 children with pertussis diagnosed by culture were admitted to Shenzhen Children's Hospital infectious Department. The median age of children in MR group was 4 (3, 6) months, which was older than that in MS group (*Z*= 4.38, *P*< 0. 05). 38 cases (48.71%) in MR group had been vaccinated, which was significantly higher than that in MS group (*χ*^2^= 7.24, *P*< 0.05). 25 cases (32.05%) in MR group had wheezing, which was higher than 12 cases (15.79%) in MS group (*χ*^2^= 5.58, *P*< 0.05). 24 cases (30.77%) in MR group were treated with gamma immunoglobulin, which was more frequently than that in 12 cases (15.79%) in MS group (*χ*^2^= 4.82, *P*< 0.05). Gamma immunoglobulin was used in 24 cases (30.77%) in MR group, which was more common than that used in 12 cases (15.79%) in MS group (*χ*^2^= 4.82, *P*< 0.05). 13 cases (16.67%) in MR group were treated with SMZ-TMP, which was more common than that in MS group (*χ*^2^= 13.8, *P*< 0.05). The logarithmic value of PCR copy number of pertussis in MR group (5.86 ± 1.07) was higher than that in MS group (*t*= 5.67, *P* < 0.05).

**Conclusion:**

The macrolides-resistance of Bordetella pertussis was common. Except wheezing, there was no significant difference in other clinical manifestations of children infected with macrolides-resistant or macrolides-sensitive Bordetella pertussis. The infection of macrolides-resistant strains was more common in children who had been vaccinated, and as to the patients infected with macrolides-resistant strains, most of them could be cured effectively with the treatment of erythromycin. Only small part of them need to be treated with SMZ-TMP.

**Disclosures:**

**All Authors**: No reported disclosures

